# Syndrome de Casse-Noisette: cause rare de douleurs abdominales chez l’adulte, à ne pas méconnaître (à propos d’un cas)

**DOI:** 10.11604/pamj.2021.38.288.28387

**Published:** 2021-03-19

**Authors:** Mohamed Reda Haboussi, Houria Tabakh, Amina Mouffak, Amine Fahl, Touda Kebbou, Najwa Touil, Abdellatif Siwane, Omar Kacimi, Nabil Chikhaoui

**Affiliations:** 1 Service de la Radiologie des Urgences, Hôpital Ibn Rochd, Casablanca, Maroc

**Keywords:** Syndrome de Casse-Noisette, veine rénale gauche, varices pelviennes, artère mésentérique supérieure, à propos d’un cas, Nutcraker syndrome, left renal vein, pelvic varices, superior mesenteric artery, case report

## Abstract

De nos jours, la connaissance des anomalies vasculaires est primordiale pour tous les spécialistes en pratique clinique et peut prévenir de graves complications suite à des interventions précoces. Le syndrome de Casse-Noisette ou Nutcraker syndrome résulte d´une compression de la veine rénale gauche (VRG), généralement dans la fourchette formée par l´aorte abdominale et l'artère mésentérique supérieure (AMS), conduisant à la sténose de la partie aorto-mésentérique de la veine rénale gauche et dilatation de sa partie distale. La symptomatologie reste dominée par des douleurs lombaires, abdominales, pelviennes et hématurie. Son diagnostic est basé essentiellement sur les moyens d´imagerie moderne (tomodensitométrie, échographie-Doppler, phlébographie) et son traitement est controversé. Nous rapportons le cas d´une femme âgée de 43 ans, admise dans le cadre d´un bilan radiologique devant des douleurs épigastriques avec pesanteur pelvienne suite à un syndrome de Casse-Noisette.

## Abstract (en)

Nowadays, the knowledge of vascular abnormalities is essential for all specialists in their clinical practice and may prevent serious complications through early interventions. Nutcraker syndrome is caused by left renal vein (LRV) compression, usually occurring in the fork formed by the abdominal aorta and the superior mesenteric artery (SMA) and leading to the narrowing of the aortomesenteric portion of the left renal vein and to the dilatation of its distal portion. Symptoms are dominated by lumbar, abdominal, pelvic pain and haematuria. Diagnosis is essentially based on modern imaging tests (computed tomography (CT) scan, Doppler-ultrasound, phlebography) and treatment is controversial. We here report the case of a 43-year-old woman, admitted with epigastric pain associated with pelvic heaviness following nutcraker syndrome requiring radiological screening on admission.

## Introduction

Le syndrome de Casse-Noisette ou Nutcraker syndrome résulte d´une compression de la veine rénale gauche (VRG), généralement dans la fourchette formée par l´aorte abdominale et l'artère mésentérique supérieure (AMS), conduisant à la sténose de la partie aorto-mésentérique de la veine rénale gauche et dilatation de sa partie distale [1]. La symptomatologie reste dominée par des douleurs lombaires, abdominales et l´hématurie. Nous rapportons le cas d´une femme de 43 ans, qui s´est présentée pour algies pelviennes d´évolution chronique, chez qui le diagnostic a été retenu devant les signes scanographiques.

## Patient et observation

**Figure 1:**
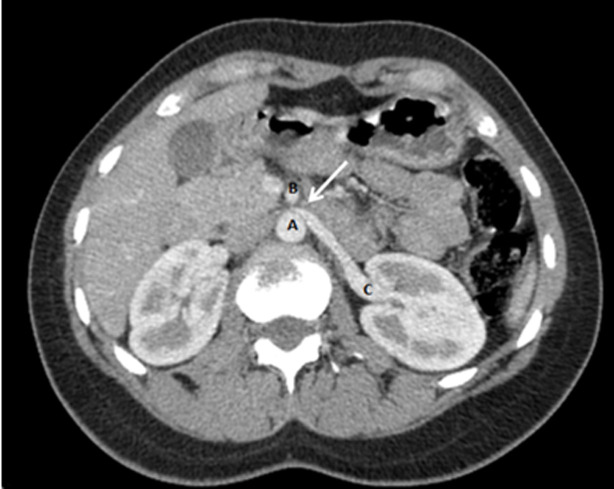
coupe axiale scanner abdomino-pelvien avant et après injection de PDC, signe du bec (flèche) qui désigne au niveau de la fourchette aorto-mésentérique; A) aorte abdominale; B) artère mésentérique supérieure; C) la compression de la veine rénale gauche; ratio diamètre portion hilaire et aorto-mésentérique de la veine rénale gauche, 6,47 (>4,9), remplissant les critères diagnostic du SCN

**Figure 2:**
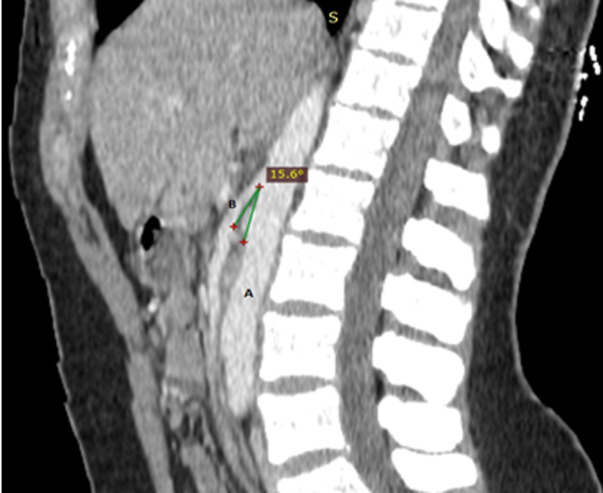
coupe sagittale scanner abdomino-pelvien avant et après injection de PDC; A) angulation entre l´aorte abdominale et l´artère mésentérique supérieure; B) moins de 41° (mesuré à 15,6°), remplissant les critères diagnostic du SCN

**Figure 3:**
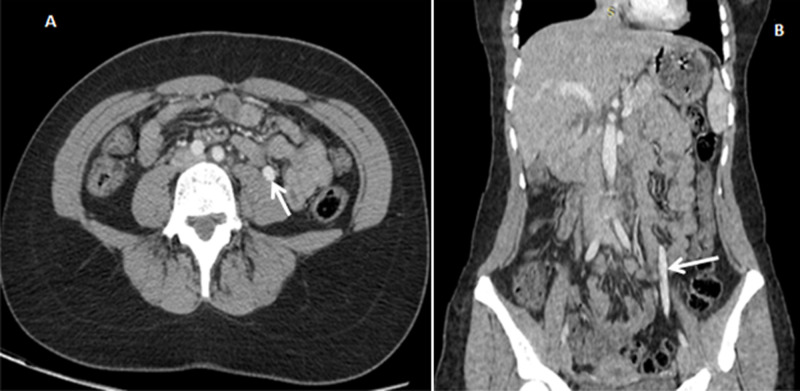
A) coupes axiale; B) coronale scanner abdomino-pelvien avant et après injection de PDC; dilatation de la veine ovarienne gauche (flèche)

**Figure 4:**
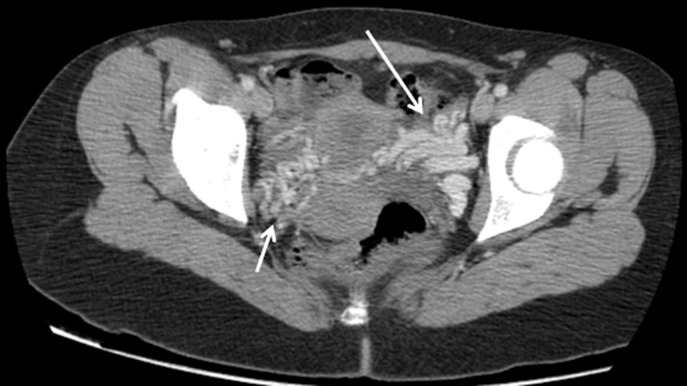
coupe axiale scanner abdomino-pelvien avant et après injection de PDC; circulations veineuses collatérales (varices pelviennes) (flèches)

Il s´agit d´une jeune femme âgée de 43 ans, sans antécédents pathologiques particuliers admise au service de radiologie pour le bilan d´une douleur abdominale de siège épigastrique paroxystique non améliorée par les antalgiques usuels, associée à une pesanteur pelvienne, évoluant depuis 3 mois. L´examen clinique était normal. Le bilan radiologique standard (échographie abdomino-pelvienne) et biologique (numération formule sanguine (NFS), lipasémie) étaient normaux. Un scanner abdomino-pelvien a été réalisé avant et après injection de produits de contraste (PDC) n´a pas objectivé de pathologie spécifique habituelle. Cependant il a mis en évidence une dilatation de la veine rénale gauche (VRG) (ratio diamètre portion hilaire et aorto-mésentérique: 6,47 (>4,9)) (Figure 1) avec rétrécissement de sa portion piégée dans la fourchette formée par l´aorte abdominale et l´artère mésentérique supérieure (AMS) dont l´angle mesure 15,6° (Figure 2), ainsi qu´une dilatation de la veine ovarienne gauche et circulations veineuses pelviennes (Figure 3 et Figure 4). Devant ce tableau scanographique très évocateur, la tomodensitométrie était suffisante pour poser le diagnostic du syndrome de Casse-Noisette, sans avoir recours à la phlébographie. Apres discussion multidisciplinaire, l´abstention thérapeutique a été indiquée vu le caractère intermittent de la symptomatologie clinique et son intensité modérée.

## Discussion

**Figure 5:**
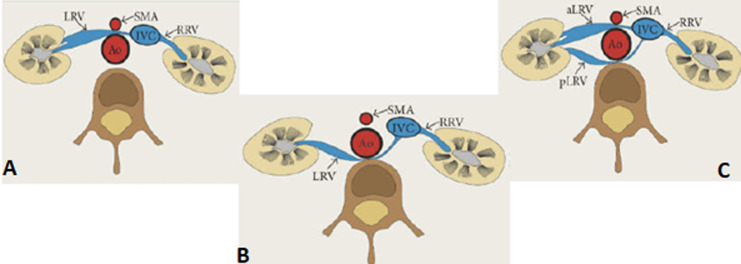
schéma anatomique montrant les différentes variantes du syndrome de Casse-Noisette; A) variante antérieure (la plus fréquente) correspond à une compression de la VRG, normalement située, par l'aorte et l´AMS; B) variante postérieure désignant la participation d´une VRG rétro-aortique, dans un petit espace entre l´aorte abdominale et la colonne vertébrale; C) variante mixte, incluant l´association des deux formes dans le cadre d´une veine rénale gauche circum-aortique ou double

Le syndrome de Casse-Noisette (SCN) se réfère à une compression de la VRG lors de son passage dans la pince aorto-mésentérique. C´est une entité rare, mais probablement sous-estimée. Sa prévalence est plus élevée chez le sujet jeune de 30 à 40 ans, avec une atteinte plus fréquente chez les femmes [1,2]. Le syndrome de piégeage peut être divisé en 3 types: antérieur, postérieur et mixte (Figure 5) [3]. Le SCN antérieur: retrouvé dans la majorité des cas, il correspond à une compression de la VRG, normalement située, par l'aorte et l´AMS; tandis que la variante postérieure, désigne la participation d´une VRG rétro-aortique, dans un petit espace entre l´aorte abdominale et la colonne vertébrale [4].

Sa physiopathologie demeure inconnue mais plusieurs hypothèses ont été avancées: variantes anatomiques [5,6]; duplicité de la veine rénale gauche, dans ce cas les patients peuvent souffrir des deux composantes antérieure et postérieure; reins ectopiques ou en fer à cheval; la naissance ectopique des artères spermatiques et ovariennes peut également étrangler la veine rénale; cofacteurs: hyperpression du réseau veineux (cave plus que portal) peuvent contribuer à la majoration ou l´apparition des signes [7]; la prédominance féminine du syndrome chez l´adulte pourrait s´expliquer par une altération valvulaire des veines gonadiques suite à des hyperpressions veineuses lors de la grossesse [7]; un seul cas d´anévrisme de l´aorte en dessous de l´AMS a été révélé parce qu´il avait induit un syndrome de Casse-Noisette. Par contre, toutes les causes de compression extrinsèques de la veine rénale peuvent induire des syndromes de Casse-Noisette secondaires, dont des cancers du pancréas, tumeurs rétropéritonéales, et adénopathies para-aortiques [6].

Les manifestations cliniques les plus courantes sont variées, induites par la stase veineuse en amont de la VRG: hématurie microscopique asymptomatique, hématurie macroscopique, douleurs abdominales ou du flanc gauche, varices pelviennes. Tous ces symptômes sont très variables et parfois difficiles à corréler avec les découvertes anatomiques: certains sujets porteurs d´une compression marquée de la VRG sont totalement asymptomatiques [8]. Le diagnostic du SCN repose essentiellement sur les moyens d´imagerie moderne. Le scanner multibarrettes, par ses acquisitions multiplanaires, offre un avantage certain pour l´établissement du diagnostic en objectivant différents critères notamment: une compression de le VRG dans l´espace formé par l´artère mésentérique supérieure et l'aorte, la distension des veines gonadiques et la congestion pelvienne. Certains auteurs ont donc tenté de valider ces critères, dont Kim *et al*. désignant [9] le signe de Bec très évocateur sur les coupes axiales (compression de la VRG dans la fourchette aorto-mésentérique) avec une spécificité de 88,9% (Figure 1); l´angulation entre l´aorte et l´AMS (<41°) avec une spécificité de 55,6% (Figure 2); le rapport de diamètre de la VRG (rapport hilaire-aorto-mésentérique) >4,9 avec une spécificité de 100% (Figure 1); distension veines gonadiques et congestion pelvienne (Figure 3 et Figure 4).

Malgré le grand apport de la tomodensitométrie dans le diagnostic du syndrome de Casse-Noisette par sa haute précision des paramètres anatomiques et son caractère non invasif, elle présente des contraintes non négligeables de nos jours tels que l´exposition aux radiations et aux risques d'allergie aux produits de contraste [6]. Si le diagnostic reste incertain, le gradient de pression entre la VRG et la veine cave inférieure (VCI) joue un rôle important dans le diagnostic du syndrome de Casse-Noisette, il est mesuré lors d´un examen Doppler mais la phlébographie reste la méthode la plus précise (tous deux demeurent le gold standard) [10]. Selon les revues de la littérature, 1 mmHg correspond au gradient de pression normal, Beinart *et al*. ont désigné qu'un gradient de pression de 1 mmHg ou plus indique une hypertension VRG [11,12]. L´échographie Doppler est une technique qui peut s´avérer très utile pour conforter le diagnostic si on arrive à montrer que le rapport entre les vitesses maximales de la VRG au niveau de la sténose et de la distension maximale d´amont est supérieur ou égal à cinq (sensibilité de 69 à 90% et spécificité de 89 à 100%) [13]. En ce qui concerne le traitement, ce dernier reste très controversé. A côté de la chirurgie (transposition de la VRG et/ou l´AMS) indiquée en cas de douleurs intenses et hématurie massive, certaines équipes réalisent un traitement endovasculaire. Sinon une abstention thérapeutique est de règle comme le cas de notre patiente (symptomatologie minime ou absente) [14].

## Conclusion

Le syndrome de Casse-Noisette est une entité rare, à évoquer dans la gamme diagnostique des étiologies rares des douleurs abdominales inexpliquées du sujet jeune, a fortiori si elles sont latéralisées à gauche et accompagnée d´hématurie. Étant donné son individualisation relativement récente, la prise en charge de ce syndrome n´est encore bien codifiée (chirurgie, endovasculaire ou abstention).

## References

[1] Takezawa K, Nakazawa S, Yoneda S, Tanigawa G, Fujita K, Okumi M (2011). Renal autotransplantation for the treatment of nutcracker phenomenon which caused varicocele rupture: a case report. Hinyokika Kiyo.

[2] Andrianne R, Limet R, Waltregny D, de Leval J (2002). Hematuria caused by nutcracker syndrome: peroperative confirmation of its presence. Progres En Urol J Assoc Francaise Urol Soc Francaise Urol.

[3] Orczyk K, Wysiadecki G, Majos A, Stefańczyk L, Topol M, Polguj M (2017). What each clinical anatomist has to know about left renal vein entrapment syndrome (nutcracker syndrome): a review of the most important findings. BioMed Res Int.

[4] Menard MT (2009). Nutcracker syndrome: when should it be treated and how. Perspect Vasc Surg Endovasc Ther.

[5] Panagar AD, Subhash RLP, Suresh BS, Nagaraj DN (2014). Circumaortic left renal vein-a rare case report. J Clin Diagn Res JCDR.

[6] Kurklinsky AK, Rooke TW (2010). Nutcracker phenomenon and nutcracker syndrome. Mayo Clin Proc.

[7] Yih NDC, Chyen LH, Cunli Y, Jaywantraj PS, Isip ABC, Anil SA (2014). Renosplenic shunting in the nutcracker phenomenon: a discussion and paradigm shift in options? A novel approach to treating nutcracker syndrome. Int J Angiol Off Publ Int Coll Angiol Inc.

[8] Berthelot J-M, Douane F, Maugars Y, Frampas E (2017). Le syndrome nutcracker: une cause rare de douleurs lombaires gauches mais aussi de souffrances pelviennes inexpliquées. Rev Rhum.

[9] Kim KW, Cho JY, Kim SH, Yoon JH, Kim DS, Chung JW (2011). Diagnostic value of computed tomographic findings of nutcracker syndrome: correlation with renal venography and renocaval pressure gradients. Eur J Radiol.

[10] Ahmed K, Sampath R, Khan MS (2006). Current trends in the diagnosis and management of renal nutcracker syndrome: a review. Eur J Vasc Endovasc Surg.

[11] Wei SM, Chen ZD, Zhou M (2003). Intravenous stent placement for treatment of the nutcracker syndrome. J Urol.

[12] Beinart C, Sniderman KW, Saddekni S, Weiner M, Vaughan Jr ED, Sos TA (1982). Left renal vein hypertension: a cause of occult hematuria. Radiology.

[13] Shin JI, Park JM, Lee JS, Kim MJ (2007). Effect of renal doppler ultrasound on the detection of nutcracker syndrome in children with hematuria. Eur J Pediatr.

[14] Kim JY, Joh JH, Choi HY, Do YS, Shin SW, Kim DI (2006). Transposition of the left renal vein in nutcracker syndrome. Eur J Vasc Endovasc Surg.

